# Ion-Induced Volume Transition in Gels and Its Role in Biology

**DOI:** 10.3390/gels7010020

**Published:** 2021-02-18

**Authors:** Matan Mussel, Peter J. Basser, Ferenc Horkay

**Affiliations:** Eunice Kennedy Shriver National Institute of Child Health and Human Development, National Institutes of Health, Bethesda, MD 20892, USA; basserp@mail.nih.gov

**Keywords:** polyelectrolyte gel, volume phase transition, biopolymers, DNA, cell secretion, xylem flow, abrupt depolarization, membraneless organelles

## Abstract

Incremental changes in ionic composition, solvent quality, and temperature can lead to reversible and abrupt structural changes in many synthetic and biopolymer systems. In the biological milieu, this nonlinear response is believed to play an important functional role in various biological systems, including DNA condensation, cell secretion, water flow in xylem of plants, cell resting potential, and formation of membraneless organelles. While these systems are markedly different from one another, a physicochemical framework that treats them as polyelectrolytes, provides a means to interpret experimental results and make in silico predictions. This article summarizes experimental results made on ion-induced volume phase transition in a polyelectrolyte model gel (sodium polyacrylate) and observations on the above-mentioned biological systems indicating the existence of a steep response.

## 1. Introduction

Charged biopolymers are ubiquitous in living systems [1]. Examples range from relatively simple solutions of charged polynucleotides and proteins to complex structures such as the cytoplasm, cell nuclei, and extracellular matrices. In particular, the association of biopolymer molecules into supramolecular assemblies through reversible noncovalent bonds plays a central role in the formation of many functional biological structures.

The molecular details are evidently important for many biological functions [2], but the perceived complexity of biological systems often challenges the formulation of quantitative models with predictive power. Accordingly, highly idealized models may provide insight into the origins of certain phenomena, even if they do not account for all of the components and their interactions. Indeed, it was repeatedly demonstrated in many physical systems, especially in the vicinity of a phase transition that universal principles and macroscopic variables govern the response of the system irrespective of many of the details [3,4]. In particular, the important role that polymer physics plays in governing the behavior of biological systems has been emphasized by many researchers, and it offers a powerful framework to identify critical variables required to describe certain biological phenomena [5,6,7].

Synthetic biomaterials designed to mimic the physiological behavior of living systems enable researchers to study the physical and chemical basis of minimal cell structures [8]. These systems are often composed of polyelectrolytes whose characteristics are largely determined by the properties of the hydrated polyelectrolyte molecules, the counterions present in the solution, and the interaction between the components (polyelectrolytes, water molecules, and counterions) [9]. There are various physical forces and interactions implicated in this clustering process, including electrostatic repulsion and attraction, hydrophobic and hydrophilic interactions, hydrogen bonding, and van der Waals forces [10].

In this article, we focus on ion-induced volume transition in polyelectrolyte gels and its potential role in governing a diverse range of phenomena in living systems. Our aims are to emphasize certain principles shared in several physiological processes, and to describe the progress that has been made in understanding ion-induced volume transition in polyelectrolyte gels. The article is organized as follows. In Section 2, we describe macroscopic observations made on a polyacrylate-based model system, with an emphasis on the effect of ion competition, a common situation in cellular dynamics. In Section 3, we describe the role of the abrupt structural response of biopolymers and the ion-exchange processes at work in five biological systems. These include (1) compaction of DNA molecules [11], (2) storage and release of secretory products [7], (3) ability to change the hydraulic resistance to water in the xylem in plants [12], (4) abrupt change in the cell resting potential upon incrementally changing the ionic composition of the extracellular solution [6], and (5) formation of membraneless organelles [13]. In Section 4, we describe minimal theoretical models that address the interactions among the polymer network, water, and ions, and may be adequate to describe the more complex biological systems. Conclusions and outlook are drawn in Section 5. Hereafter, we use the nomenclature that ∼means *of the order of*, and ≈means *approximately*.

## 2. Ion-Induced Volume Transition

**Figure 1:**
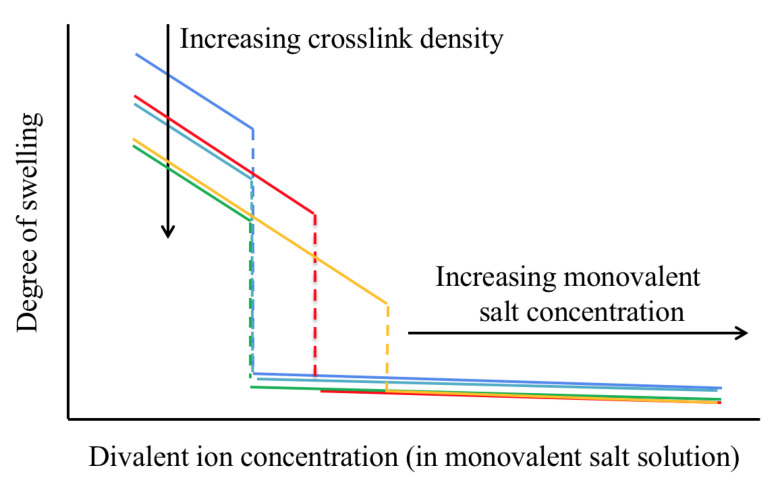
Schematic representation of the variation of the equilibrium degree of swelling of polyelectrolyte gels as a function of the concentration of divalent salt in the external bath solution. Additionally shown are the effects of increasing the crosslink density (blue-light blue-green curves) and monovalent salt concentration (blue-red-orange curves) [17].

The rationale of this paper is that despite important compositional, structural, and morphological differences, weakly cross-linked, highly charged polyelectrolyte gels share certain characteristics, especially (but not only) near the volume phase transition [14]. Specifically, in an aqueous solution containing only a monovalent salt (e.g., NaCl and KCl), the degree of swelling of many polyelectrolyte gels can be very large, ∼100–1000, due to the electrostatic repulsion between the charged sites on the polymer backbone. As the concentration of a divalent salt (e.g., CaCl${}_{2}$ and SrCl${}_{2}$) is increased in the solution, the degree of swelling decreases (Figure 1). At a critical concentration ratio of the divalent to monovalent salt, ≈1–10%, the gel collapses [15,16].

In the following section, we briefly summarize important results obtained from systematic investigations of the macroscopic properties of *polyacrylate* gels. These include the degree of swelling in different ionic environments, elastic modulus, NMR parameters, electric potential difference, and ion partitioning. Our purpose is to illustrate the richness of the phenomenon, and to provide insights into possible behavior, which is more challenging to measure in biological systems as described in Section 3 below.

**Figure 2:**
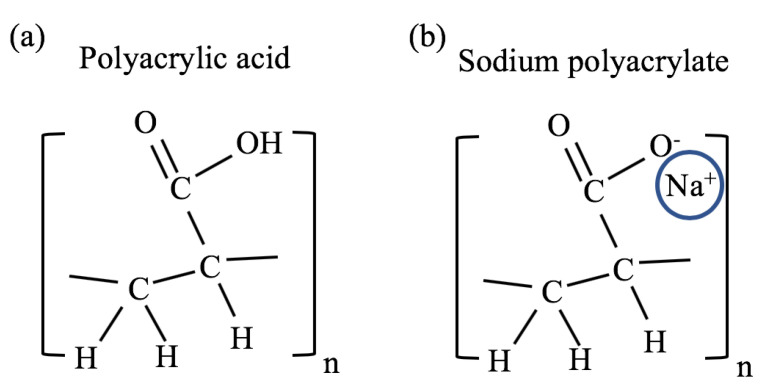
Chemical structure of (**a**) polyacrylic acid and (**b**) sodium polyacrylate.

The major advantages of the polyacrylate gel model are that it is one of the simplest and most investigated polyelectrolyte gel system, it has a long shelf life, is easy to work with, and is inexpensive. The chemical structure of polyacrylate is composed of repeating vinyl groups containing a carboxylate anion. The chemical structure of the acidic form (*polyacrylic acid*) is shown in Figure 2a, where a hydrogen ion is attached to the carboxylate group. In sodium polyacrylate (NaPA), the hydrogen ion is replaced by a sodium ion as shown in Figure 2b.

### 2.1. Degree of Swelling

#### 2.1.1. Effect of Monovalent Counterions on Gel Swelling

The pKa value of carboxylic acid is ≈4.55 at room temperature and pressure conditions [18]. This means that when a polyacrylic acid gel is immersed in water, the concentration of dissociated hydrogen ions satisfies

(1) $$\frac{\left[H^{+}\right]\left[COO^{-}\right]}{\left[COOH\right]}=10^{-4.55}\;\mathrm{mol}/L$$

which is very small. Such system is called weak acid as the hydrogen ions are strongly associated with the polymer backbone [19]. The (mostly) uncharged cross-linked polyacrylic acid gel has a degree of swelling of ≈2–10 at room temperature, which depends on additional parameters such as the cross-link density [20].

A significant change in the swelling degree of this gel occurs when some of the hydrogen ions associated with the carboxylate anions are replaced with alkali metal ions; Li${}^{+}$, Na${}^{+}$, K${}^{+}$, etc. (Figure 2b). This is obtained by neutralizing the acid form using alkaline hydroxides (e.g., LiOH, NaOH, and KOH). The hydroxides compete with the carboxylate groups for the hydrogen ions, and the alkali metal ions replace the hydrogen ions. This neutralization process is favorable because the base dissociation constant (pKb) of these Arrhenius bases is low (<1) while the pKa of water is high (≈14) [21]. Hydrogen ions associated with the vinyl group of the polymer hardly disassociate in this process because their pKa is significantly higher (>40) [22].

When monovalent cations directly interact with the carboxylate anions, the ionic cloud is relatively extended (∼1 nm), which can be estimated by the Poisson–Boltzmann model [23,24]. The electrostatic repulsion between the polymer chains results in a highly swollen gel at equilibrium; the highest degree of swelling can reach values of ≈500–1000 in pure water [15,16].

When exposed to excess monovalent salt, the equilibrium degree of swelling of polyacrylate gels, *q*, was empirically found to exhibit a power-law decrease as the salt concentration, *c*, increases in the external bath solution, $q\propto c^{-n}$ [15]. The experimental value of the exponent *n* is typically smaller than 1, which is consistent with the theoretical value n=0.6 [25]. However, *n* depends on multiple properties of the gel and its environment, including cross-link density and temperature [26]. Experimentally, for a weakly cross-linked polyacrylate gel n≈0.5 was reported [16]. Using different alkali metal ions, the dependencies of the degrees of swelling on the ion concentrations are not significantly different, implying that the main effect of monovalent cations is to screen the electrostatic repulsion among the anionic groups on the polyacrylate chains [16].

#### 2.1.2. Effect of Multivalent Counterions on Gel Swelling

**Figure 3:**
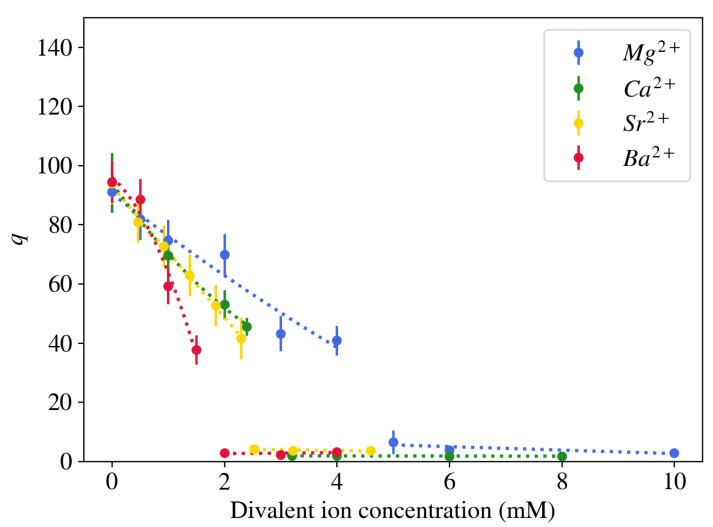
Comparison of the effect of different alkaline earth metal ions on the degree of swelling of NaPA gel brought in equilibrium with an aqueous solution containing 40 mM NaCl at pH = 5.5.

In contrast to monovalent counterions, multivalent cations have significantly stronger affinity to the negatively charged sites on the polymer molecule. As a result of the larger electrical charge, these cations typically occupy a denser and more adjacent layer near the polyelectrolyte chains [23,27]. When a neutralized polyelectrolyte hydrogel is placed in a salt solution that contains a mixture of mono- and divalent ions, the competition between the ions results in a partially deswollen state (Figure 1). Because of their strong affinity to the polymer network, significantly fewer divalent counterions in the surrounding liquid are needed to reduce the volume of the gel as compared to monovalent counterions. Moreover, above a critical concentration the polymer chains collapse and the majority of solvent is expelled from the gel (q≈ 2–3) [15]. No significant change in the gel volume is further observed as the divalent salt concentration increases. In contrast to alkali metal ions, alkaline earth metal ions differ in their effect on the equilibrium degree of swelling of NaPA gels. Figure 3 shows that divalent cations with larger atomic mass induce gel collapse at lower concentrations. Higher-valence counterions affect the degree of swelling of the gel in a similar way as divalent counterions, but the transition point is shifted towards lower cation concentration [28].

The effect of divalent cations on the degree of swelling depends on additional parameters, including the cross-link density, concentration of the ionized groups of the network chains, concentration of the monovalent salt in the surrounding solution, and temperature [17]. These are important effects that may be related to abnormalities in biological systems [29]. In particular, in polyacrylate gels increasing the cross-link density does not modify the critical divalent cation concentration at which the volume transition occurs, but significantly reduces the degree of swelling in the swollen phase (blue-light blue-green curves in Figure 1). The latter behavior results from the decrease in the average chain length. As the concentration of charged groups on the polymer chains is reduced, the difference between the degree of swelling in the swollen and collapsed states gradually decreases. Increasing the concentration of the monovalent salt in the bath solution requires higher divalent ion concentration to induce volume transition (blue-red-orange curves in Figure 1). A large excess of monovalent salt as compared to divalent salt can eventually prevent discontinuous volume transition. As the temperature increases, the degree of swelling decreases and volume transition occurs at lower divalent salt concentrations [17]. Furthermore, decreasing the pH of the solution shows a similar effect as increasing the concentration of the divalent salt, i.e., volume transition can be induced by increasing the concentration of hydrogen ions in the solution [30]. This response is explained by the strong affinity of the hydrogen ions to the charged groups of the polymer chains [31].

In several previous studies made on polyacrylate solutions and gels, multivalent cations were assumed to form cross-links. However, addition of divalent cations (e.g., Ca${}^{2+}$ ions) to the external bath solution has a negligible effect on the elastic modulus of polyacrylate gels [15]. Additional and independent arguments supporting this result include (1) changing the cross-link density of polyacrylate gels does not affect the critical divalent ion concentration required to induce volume transition [17]. (2) SAXS or SANS measurements at high values of the scattering vector make it possible to detect local structural changes of the system. While chemical cross-linking generates large-scale inhomogeneities, increasing the divalent salt concentration does not produce similar changes in the scattering profiles [32]. In these experiments, gels were prepared with ∼0.001–0.01 molar ratios of cross-linker (N,N′-methylenebisacrylamide) to monomer units, and ∼0.01–1 calcium ions per monomer unit [15,16]. (3) Decreasing the pH has a similar effect on the degree of swelling, as increasing the concentration of divalent cations even though hydrogen ions are monovalent [30].

### 2.2. NMR Parameters

Nuclear Magnetic Resonance (NMR) is an efficient method to characterize properties of soft materials by measuring the interaction of nuclear spins in the presence of a strong magnetic field, for instance, hydrogen (${}^{1}H$), carbon (${}^{13}C$), or sodium (${}^{23}Na$). The three most common parameters extracted from NMR measurements are the spin–lattice relaxation rate ($R_{1}$), the spin–spin relaxation rate ($R_{2}$), and the apparent diffusion coefficient (ADC). The spin–lattice relaxation is typically the result of thermal motion of nuclei in the sample, and the spin–spin relaxation is caused by spin–spin interactions between the nuclei that induce local magnetic field inhomogeneities. The relaxation parameters are governed by molecular fluctuations quantified by the averaged rotational correlation time of the molecules, $\tau_{c}$ [33]. The ADC measures the diffusion coefficient of molecules whose nuclei are spin-labeled [34]. All three parameters are sensitive to the presence of polymer networks, and, in particular, abruptly change their values at the volume transition [35].

The solvent nuclei in a salt solution interacting with a polymer network can be crudely divided into two subgroups: one is those that are strongly interacting with the polymer chains and the other is only weakly interacting with the chains [36]. It is mainly the first layer of solvent molecules around the polymer chains that interacts with the polymers, and $R_{1}$ and $R_{2}$ are dominated by this strongly interacting population [37]. The rotational correlation time of the strongly interacting water molecules is ∼1 ns, while for the “free” solvent it is ∼1 ps. Measurements of ion-induced volume transition in polyacrylate gels suggest that the correlation time of the strongly interacting water compartment increases monotonically upon increasing the divalent salt concentration in the external bath solution. This is an indication that the mean water–polymer dispersive interaction is influenced by the ionic environment. A similar trend was observed for sodium ions interacting with polyacrylate chains [37]. The ADC in swollen gels is only slightly smaller than that in free water, because the polymer volume fraction is generally small, <1%, and mostly what is being observed is the diffusion of the free water phase. In contrast, in collapsed gels the value of the ADC decreases to about one-third of its value in the swollen state, because the polymer volume fraction is >20% and the diffusion of solvent molecules is significantly hindered by the polymer chains [37].

### 2.3. Electric Potential Difference and Ion Partitioning

Polyelectrolyte gels contain fixed charged sites on the network chains as well as mobile ions in the aqueous solution. While the gel is electrically neutral, the competition between electrostatic interactions and ionic diffusion results in an electric double layer at the polymer/water interface. As a result, an electric potential difference can be measured between the gel interior and the external bath solution with an order of magnitude $∼k_{B}T/e\approx 1-100$ mV. For anionic gels, the potential difference is negative, while for cationic gels, it is positive. In dilute salt solutions, the measured electric potential difference is in agreement with the theory of the Donnan potential. These properties were measured, for instance, in polyacrylamide/polyacrylic acid [38], poly(vinyl alcohol-co-2-acrylamido-2-methyl propane sulfonic acid) [39], and 2-Acrylamido-2-methyl propane sulfonic acid and Acryloyloxy-ethyl-trimethylammonium chloride gels [40].

**Figure 4:**
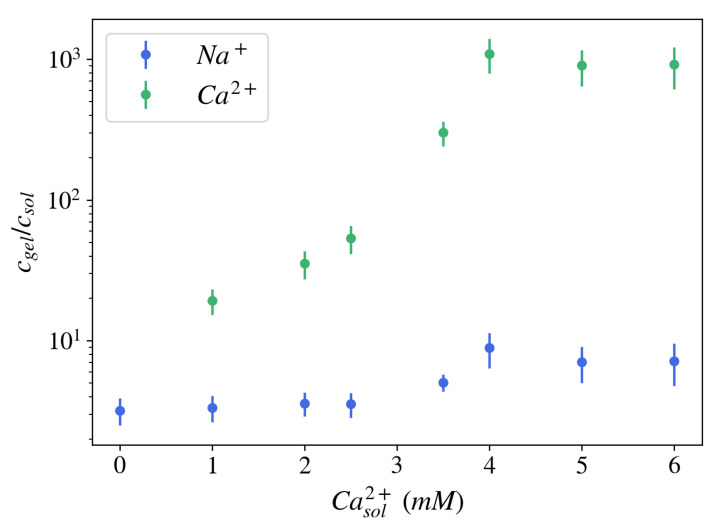
Ion partition coefficient measured for NaPA gels brought in equilibrium with an aqueous solution at pH = 5.5, containing 40 mM NaCl, and different concentrations of CaCl${}_{2}$. HNO${}_{3}$ was used to extract the ions from the gel. The ion concentration was determined by Inductively Coupled Plasma Optical Emission Spectrometry (ICP-OES) both in gels and solutions.

The formation of an electric potential difference is closely related to ionic partitioning in the gel, namely the ratio of ionic concentrations in the gel vs. in the equilibrium bath solution, $c_{j}^{gel}/c_{j}^{sol}$ ($j=Na^{+},Cl^{-},Ca^{2+}\ldots$). For NaPA gels brought to equilibrium with a solution at pH = 5.5, containing 40 mM NaCl, and different concentrations of CaCl${}_{2}$, the ion partitioning coefficient for sodium ions was found to be 2–10, i.e., the concentration of sodium ions was 2–10 times greater inside the gel than in the surrounding free solution (Figure 4). For calcium ions, the ion partition coefficient is substantially larger: 10–1000. Both ion partition and ion exchange depend on environmental parameters such as pH and temperature, and specific interactions between ions and polyelectrolyte molecules (ion selectivity) may also play a role [41].

## 3. Evidence of Ion-Induced Abrupt Transition in Biological Systems

In this section, we describe five cellular processes in which a steep response induced by ion-exchange, along with other environmental parameters, is suggested to play an important functional role. Given the diversity of changes occurring in NaPA gels near volume transition, it is not unlikely that related behavior happens also in these complicated biological systems; for instance, abrupt changes in the swelling degree, elastic modulus, ionic concentrations inside the gel, values of the NMR relaxation parameters, local electric fields, and diffusion of water and ions.

### 3.1. The Compaction Process of DNA Molecules

DNA is a semi-flexible polymer forming a double helix. Each pair of nucleotides carries two negatively charged phosphate groups, making the DNA molecule a highly charged polyelectrolyte with a hydrophobic backbone due to the lack of charge in the base of the nucleotides [42]. Specifically, when dissolved in monovalent salt solution, DNA stretches to a coil conformation. This is mainly the result of the electrostatic repulsion between the negatively charged phosphate groups [42]. In contrast, the interaction of DNA molecules with multivalent cations leads to significant compaction [11,42,43]. DNA equilibrium conformation depends on the balance of entropic, electrostatic, and dispersive forces, as well as other factors, including counterion size, hydrophobic regions, and self-assembly of the stiff DNA molecules [42,44]. These competing forces result in a variety of equilibrium conformations of collapsed or partially collapsed DNA chains, including toroid, rod, spherical globule, flowers, and racket-shaped condensates [42].

**Figure 5:**
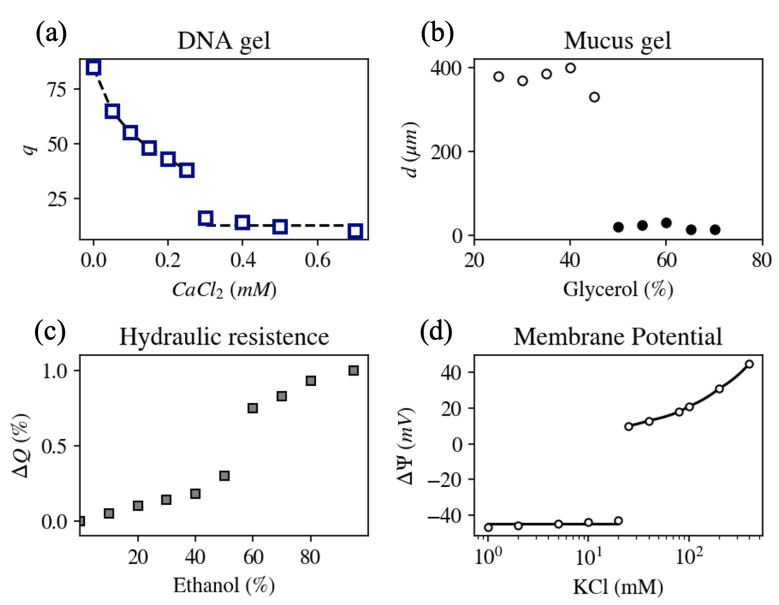
(**a**) Variation of the equilibrium degree of swelling of DNA gels as a function of CaCl${}_{2}$ concentration. In addition to the divalent salt, the solution contains 40 mM NaCl [45]. (**b**) Diameter, *d*, of mucus extracted from a giant secretory granule of the terrestrial slug *Ariolimax columbianus* as a function of glycerol/water ratio at pH = 7 [7]. (**c**) Relative change in flow rate, ΔQ, in a stem of *Laurus nobilis* as a function of ethanol concentration (solvent quality) [12]. (**d**) Equilibrium membrane potential, ΔΨ, in an internally perfused axon as a function of KCl concentration in the extracellular solution. Abrupt depolarization is observed at 20 mM KCl [6].

Despite the apparent complexity of the DNA molecule, the response of swelling and structural properties of DNA gels to mono/divalent ion exchange was found to be similar to the response of NaPA hydrogels (Figure 5a) [45]. Multivalent cations whose charge is ≥3 in aqueous solution (e.g., as commonly found inside cells) condense DNA molecules at smaller concentrations as compared to divalent cations [11], similar to NaPa gels [28]. Furthermore, small-angle neutron scattering intensity profiles did not reveal significant differences between NaPA and DNA gels over a wide range of length scales [30,45,46]. A qualitatively similar response of these two polymers to changes in pH was also demonstrated [47]. These findings imply that in spite of differences in the chemical composition and flexibility of polyacrylate and DNA molecules, the physical forces that govern their hierarchical organization are similar. Moreover, the regulation of the rate at which specific target genes are expressed was shown to be associated with the formation of complexes with specialized proteins (transcription factors and co-activators) that interact with specific sites on the DNA chain and phase separate it from the nucleoplasm [48,49].

### 3.2. Storage and Release of Secretory Products

Many secretory cells produce and pack dense polyelectrolyte gels in vesicles made of a lipid bilayer. These vesicles remain stored inside the cell until an external or internal stimulation (e.g., calcium ions, pH, and electric current) leads to vesicle fusion into the cell membrane, and the consequent ejection of the gel into the extracellular milieu [7]. Upon contact with the extracellular space, the polyelectrolyte gel rapidly increases its volume (∼100-fold within seconds). Examples of secretory cells and the polyelectrolyte gel they produce include (1) goblet cells located in the lungs and gut of vertebrates, which secrete mucus to protect from physical, chemical, and bacterial injuries [50]; (2) mast cells present in connective tissues of many vertebrates, which release inflammatory mediator molecules such as histamine, serotonin, and heparin [51,52]; (3) chromaffin cells located in the adrenal glands of mammals that secret hormones (e.g., catecholamines such as dopamine and norepinephrine) into the bloodstream [53]; (4) mucous cells located at the body surface of terrestrial mollusks that secrete mucins either as transparent fluid or as a dense, opaque, and sticky fluid to remove toxic materials, for lubrication and moistening, and also as a defense mechanism [54]; and (5) single-cell organisms (e.g., pathogenic protozoa such as *Plasmodium* and *Toxoplasma gondii*, as well as non-parasitic protists such as *Paramecium tetraurelia* and *Tetrahymena thermophila*) that release different peptides for various purposes such as hunting, protection from predators, and adhesion [55].

Mucin, for example, is an elongated glycoprotein that can reach up to several micrometers in length and has a bottlebrush structure with a linear peptide backbone and polysaccharide side chains [56]. The side chains are negatively charged by sialic and sulfated sugar residues. As a result of the electrostatic repulsion, dissolved mucins stretch when neutralized by monovalent counterions and form a highly swollen gel (mucus). In contrast, when exposed to divalent cations, such as calcium, the mucin fibers collapse into a densely packed gel and phase separate from the solvent. This is the method used by goblet cells, for example, to pack mucus within lipid vesicles called granules.

Figure 5b shows the steady state diameter of mucus extracted from a giant secretory granule of the terrestrial slug as a function of the solvent quality (glycerol/water ratio) at pH = 7. A reversible transition from swollen (diameter > 20 μm) to collapsed state (diameter < 20 μm) was found to be either continuous or sharp, depending on various environmental properties such as pH, solvent quality, temperature, and mono- to divalent cation concentration ratio [7].

A similar behavior takes place in mast cells that store inflammatory mediator molecules (e.g., histamine and heparin). These polyelectrolytes are densely packed inside lipid vesicles by using multivalent peptides of opposite charge, forming small-volume complexes and containing almost no water [51,57]. Upon exposure to a monovalent salt solution, ion-exchange occurs and the gel rapidly swells.

Deciphering the governing principles of secretory action can be especially useful to understand a variety of diseases in which abnormal polymer hydration and ion-exchange properties are essential factors [58]. For example, cystic fibrosis is an inherited disorder that causes severe damage to the lungs and the digestive system. Cystic fibrosis mucins are thick and more viscous than normal mucin gels. Their rheological properties and swelling kinetics are abnormal, making it difficult to clear from the lungs, intestine, pancreas, and sweat glands [59]. A comprehensive model that combines competing electrical, chemical, mechanical, and thermodynamic forces acting on the gel demonstrates how the drastic increase in calcium binding affinity and increased calcium ion concentration in the liquid surrounding the mucus may cause abnormal swelling kinetics [29].

### 3.3. The Ability to Change the Hydraulic Resistance of Xylem in Plants

Long-distance water transport in land plants occurs through the xylem, an efficient network of microchannels (diameter < 500 μm) that connect the roots, stem, branches, twigs, petioles, and leaf veins. Water passes through many thousands of conduits along its way. Interconduit connections for water passage, called *pits*, enable water flow through common walls, while preventing the spread of gas bubbles as well as pathogens [60,61]. Rapid and reversible changes in the hydraulic pressure are associated with changes in the composition of salt in the xylem sap, and can alter hydraulic conductivity. For example, in perfused branch segments of *Laurus nobilis* trees the flow rate in xylem was increased by up to 2.5 times as the concentration of KCl increased from 0 to 50 mM [12].

It has been suggested that pectin polysaccharide gel, a major component of the pits, is associated with ion-mediated hydraulic changes in the xylem [12,62]. Pectins behave like anionic polyelectrolytes that can reversibly switch from swollen to collapsed state when alkali metal ions are replaced with multivalent or hydrogen cations [63]. Cation-mediated volume changes of pit membrane pectins modify the diameter of the nanometer-sized pores of pits and thus change their hydraulic conductance. Indeed, abrupt change in the flow rate was demonstrated upon gradual changes in solution ion concentration, pH, and solvent quality (Figure 5c) [12].

### 3.4. Abrupt Depolarization and the Cell Resting Potential

The electric potential inside living cells is in most cases negative as compared to the extracellular space, with resting potential values ranging between −250 and 0 mV in different cells (A counter example is the *cochlear fibrocyte*, located in the mammalian cochlea, that possesses a positive resting potential [64]) [65,66]. The resting potential, however, is not a fixed value, but depends on environmental parameters, such as the ionic content in the extracellular environment [67]. In particular, upon gradually varying the ratio of mono- to divalent cation concentrations in the extracellular solution, an abrupt increase in the resting potential is measured at a critical ionic concentration ratio. This phenomenon is called *abrupt depolarization* and was observed in several types of cells, including squid giant axon, toad myelinated nerve fiber, and internodal cells of *Characean* algae [68,69,70,71]. Figure 5d shows the equilibrium membrane potential measured in an axon that was internally perfused with a solution containing 30 mM NaF, and the extracellular solution containing 100 mM CaCl${}_{2}$ with varying concentrations of KCl [6]. At small monovalent salt concentrations (<20 mM), the resting potential was insensitive to the increase in the external potassium chloride concentration. An abrupt depolarization was obtained at a KCl concentration of 20 mM. At higher concentrations the membrane potential became sensitive to the external KCl concentration. Neither the Goldman–Hodgkin–Katz equation that describes the resting potential of cells, nor the Hodgkin–Huxley model of action potential can explain the observed sudden increase in the membrane potential difference.

The qualitative similarity in response of the resting potential in living cells (Figure 5d) and the degree of swelling in polyelectrolyte gels (Figure 1) to the ratio of mono- to divalent cation concentration led to the conjecture that polyelectrolyte gels might play an important role in the measured resting potential [6,72,73]. Indeed, an electric potential difference is associated with polyelectrolytes (Section 2.3), and the intracellular milieu is abundant with charged polymers, for example, cytoskeletal filaments [74]. Experiments focusing on the cortical layer, which is made of cytoskeletal filaments and is located below and adjacent to the lipid membrane indicated that structural changes in the layer were coupled to transient changes in the electric potential difference [75]. Furthermore, damage to the cortical layer, e.g., by using degradative enzymes, modifies the value of the resting potential and disrupts the ability of the cell to produce action potentials [76].

### 3.5. Formation of Membraneless Organelles

Compartmentalization and the establishment of intracellular heterogeneity are important aspects of cellular organization, allowing different molecular contents to regulate diverse chemical processes and biological functions that take place inside living cells. Some of the cellular compartments are organized into membrane-bound organelles (e.g., mitochondria and endoplasmic reticulum). However, other compartments contain highly concentrated assemblies of different proteins and RNA molecules (RNAs) without being encapsulated within lipid membranes.

These compartments are called *biomolecular condensates* or *membraneless organelles* [77,78]. They are formed via phase separation from the liquid-like environment, enriched in specific macromolecules that become relatively deficient in the surrounding fluid [79]. Membraneless organelles can be found in a variety of phases, including liquid droplets, hydrogels, and ordered solid-state assemblies [80]. A partial list includes germ granules [77], stress granules [81], and processing bodies [82] in the cytoplasm, as well as nucleoli [83,84], Cajal bodies [85], and nuclear speckles [86] in the cell nucleus. Each of these organelles has a distinct composition of RNAs and proteins, which is defined by their specific structure and interactions. The RNAs and proteins play an important role in the specialized function of the organelle, which may include storage, splicing, decapping, and degradation [87].

**Figure 6:**
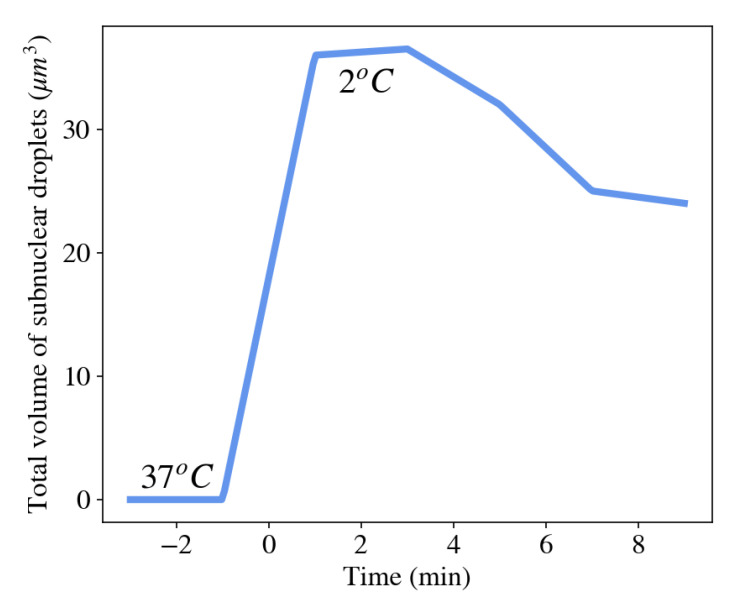
Abrupt condensation of membraneless organelles following a cold shock (2 °C). [90].

Stress granules, for example, appear in the cytoplasm of many cells shortly after the cell is exposed to environmental stresses such as thermal shock, oxidative conditions, glucose deprivation, osmotic stress, or UV irradiation [88]. The function of the formation of stress granules is believed to assemble cellular mRNAs and their associated RNA-binding proteins in order to limit their translation. This allows the cell to focus on producing essential proteins that are required for survival [89]. Figure 6 shows an abrupt increase in the total volume of condensed droplets made of Ddx4 proteins (a primary component of various membraneless organelles) as the surrounding temperature was rapidly decreased from 37 °C to 2 °C [90]. As the temperature of the cell was subsequently increased, the total volume of membraneless organelles decreased as proteins dissociated from the organelles and dispersed into the nucleoplasm.

In many cases, membraneless organelles are held by specific interactions, e.g., a particular protein binds to a particular sequence of another protein or RNA [91]. Still, the formation of membraneless organelles is distinct from aggregation that uses chemical energy to assemble and is more similar to principles of phase separation. The main difference is that membraneless organelle formation is reversible, while active aggregates use irreversible enzymatic reactions. The reversible nature of formation of membraneless organelles was demonstrated, for example, in the nucleoli by exposing the cell to oscillations of temperature, which resulted in reversible formation and disappearance of the nucleoli organelles [92].

The thermodynamic nature, response to a variety of environmental stimuli, and abundance in many cells, make polymer physics a promising framework to describe the macroscopic phase space of membraneless organelles [79,93,94]. In particular, it was suggested that the valence of the interacting particles plays an important role in formation of membraneless organelles [94,95]. However, further research is required to explore the multidimensional phase diagram of organelle formation as well as their physical properties (e.g., viscosity and elastic modulus) [79].

## 4. Theoretical Approaches to Quantify Gel-Related Biological Aspects

In the previous section, we described five unrelated biological functions whose mechanisms are based on the same underlying physical principle, namely, the nonlinear response of charged polymers to various stimuli resulting in two distinct macroscopic phases: dissolved in- and phase separated from the surrounding fluid. Although advanced physical models (Flory model, scaling theory, etc.) have been developed to describe phase transition in polyelectrolyte gels [96,97,98,99], these models cannot be easily applied to complex biological systems. The molecular details are evidently crucial in all of these systems, so each function is different from the others. Nevertheless, a universal framework offers language and tools that provide not only intuition about possible effects (e.g., how monovalent cations can disrupt the adsorption of divalent cations onto the polymer network, thus triggering gel swelling) but also makes predictions about possible changes not measured previously (e.g., changes in the electric potential, hydraulic and osmotic pressure, ion partitioning, etc.). Therefore, theoretical tools successfully describing nonliving polyelectrolyte systems may be instrumental in modeling the above-mentioned biological systems.

When choosing an appropriate model, a built-in tension exists between coarse-graining and implementing more details. The latter ensures a potential for greater accuracy for the simulation, and therefore should be favored in principle. However, this approach has several disadvantages: (1) The simulation is expensive in terms of time and computational cost. (2) Many parameters are required, which might not be accessible or measurable; as a result one may resort to the slippery slope of adding fit parameters [100]. (3) Including many details may impede us from understanding the underlying processes; i.e., to see the forest from the trees.

Opting for coarse-graining can significantly simplify the picture and is useful to cover larger regions of phase space using less time and computational resources. However, deriving macroscopic equations from microscopic details is challenging, and in the process of coarse-graining, salient parameters and phenomena may be overlooked. Furthermore, the numerical scheme required to solve the potentially nonlinear and coupled equations might be highly nontrivial.

Atomistic or coarse-grained single-particle models use quantum mechanical or classical equations of motion, respectively, to solve the dynamics of discrete entities, and provide a microscopic interpretation for certain properties of the material [101,102,103,104]. Typical periods of time and length scales investigated by single-particle models range between $10^{-12}$ and $10^{-3}$ s, and $10^{-12}$ and $10^{-3}$ m, respectively. Single-particle models are useful to describe many important aspects in biological systems. A few examples related to the systems described in Section 3, are (1) the interaction between two parallel DNA molecules in the presence of multivalent counterions [105], (2) the diffusion of nanoparticles through mucus layer [106], and (3) local conformations of pectin polysaccharide in the presence of divalent cations [107]. However, large-scale modeling that includes cooperative behavior in the gel system is challenging in the context of single-particle models.

In contrast to single-particle models, continuum mean-field models describe the properties of polyelectrolyte gels using continuous functions characterizing the mean value of density, ρ, velocity, $\vec{v}$, and internal energy, ε, at each infinitesimal point of space and time. These characteristics are separately defined for the polymer network (*p*), solvent (*s*), and additional particles involved, for instance, small ionic species ($H^{+},OH^{-},Na^{+}$, etc.) Additional variables include thermodynamic quantities, such as temperature and thermal conductivity, and forces written as gradients in hydrostatic pressure, chemical potential, electrical potential, etc. The nonlinear coupling of the various fields is dictated by the conservation of mass, momentum, and energy of the system components [108]:

(2) $$\begin{array}{ccc} \partial_{t}\rho_{j}+\nabla \cdot \left(\rho_{j}{\vec{v}}_{j}\right) & = & \sum_{i}\left(R_{ij}-R_{ji}\right) \end{array}$$

(3) $$\begin{array}{ccc} \rho_{j}\left[\partial_{t}{\vec{v}}_{j}+\left({\vec{v}}_{j}\cdot \nabla \right){\vec{v}}_{j}\right] & = & -\nabla p+\nabla \cdot \sigma_{j}+\nabla \mu_{j}+\rho_{j}{\vec{f}}_{j} \end{array}$$

(4) $$\begin{array}{ccc} \rho_{j}\left[\partial_{t}\varepsilon_{j}+\left({\vec{v}}_{j}\cdot \nabla \right)\varepsilon_{j}\right] & = & \rho_{j}\dot{q}+\nabla \left(k\nabla T\right)+\nabla \cdot \left[\left(-p+\sigma_{j}+\mu_{j}\right){\vec{v}}_{j}\right]+\rho_{j}{\vec{f}}_{j}\cdot {\vec{v}}_{j} \end{array}$$

where $R_{ij}$ represents the rate of conversion of particle type *i* into particle type *j* (e.g., when chemical reactions or adsorption processes are involved), *p* is the hydrostatic pressure, $\sigma_{j}$ the stress tensor, $\mu_{j}$ the chemical potential, ${\vec{f}}_{j}$ represents body forces such as the electric field ($-z_{j}\nabla \psi_{e}$, with $z_{j}$ valence and $\psi_{e}$ electric potential), $\dot{q}$ is the rate of volumetric heat addition per unit mass, *k* the thermal conductivity, and *T* the temperature.

The mean-field model is completed by a set of constitutive laws that define important relationships between the variables of the system. These include, for instance, the stress tensor and chemical potential of the polymer network, solvent, and ions. The choice of the constitutive relations is crucial to obtain an accurate description of the system, and represents an important source of differences between various mean-field models [109,110,111,112,113,114,115,116]. The constitutive relations are either obtained phenomenologically from experimental data or derived from first principles of polymer science and electrochemistry.

**Figure 7:**
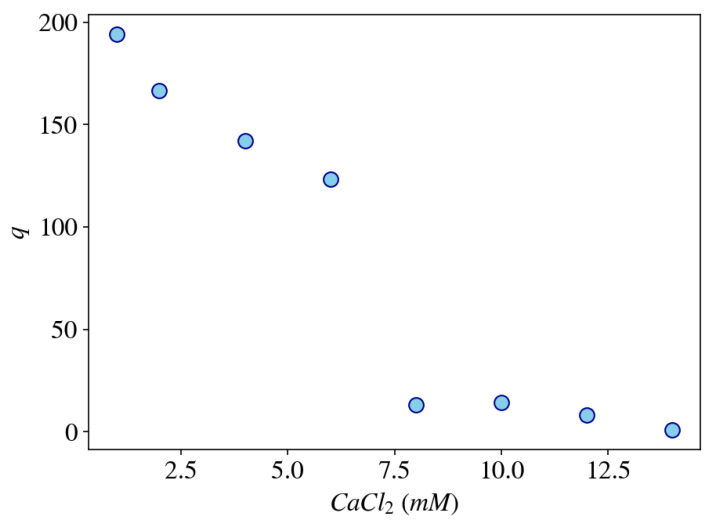
Calculation of the equilibrium degree of swelling of a gel, *q*, using a three-phase model as a function of the concentration of CaCl${}_{2}$ in the external bath solution which contains 40 mM NaCl at pH = 7.

The advantage of using mean-field models in biological systems is that they can describe the non-equilibrium dynamics of the coupled components (polymer–solvent–ions) over a large range of time and length scales (>$10^{-5}$ s and >$10^{-7}$ m), which are appropriate to describe many biological phenomena. Furthermore, the macroscopic nature of these models requires relatively few and physically well-defined macroscopic parameters (e.g., viscosity, elastic modulus, thermal conductivity, heat capacity, and diffusion coefficient). As an example, Figure 7 shows the equilibrium degree of swelling of a gel model as a function of the CaCl${}_{2}$ concentration in the external solution calculated by using a dynamic mean-field model describing a dry (unswollen) polyelectrolyte gel (e.g., weakly cross-linked polyacrylate or DNA gels) immersed in a solution containing both mono- and divalent salts (e.g., sodium chloride and calcium chloride). The model equations are based on the work of Lewis, Keener, and Fogelson [117] with minor modifications (for instance, including an elastic component) and will be described in more details in a future publication.

Despite challenges in choosing the appropriate constitutive relations, estimating the values of model parameters, and employing a numerical scheme that solves the coupled nonlinear partial differential equations, continuous multiphasic models were investigated for several biological systems. A partial list includes (1) swelling dynamics of mucus gels, which considers the effect of monovalent–divalent cation exchange on the swelling behavior [29,31,117]; (2) ability of mucus layer in the stomach to maintain pH gradient between the lumen and the stomach epithelium to protect the latter from degradation [118]; (3) analysis of ATP-driven chromatin chains (DNA molecules wrap around histone octamers) embedded in nucleoplasmic liquid [119]; (4) load-bearing mechanisms of articular cartilage [120]; (5) polymerization/depolymerization cycles induced by pH gradients that generate cell motility across a surface [121]; (6) biomass redistribution within microbial biofilms [122]; (7) mechanical processes in neutrophils [123,124]; and (8) growth of avascular tumors [125,126]. Phase separation of membraneless organelles was modeled using a similar mean-field approach, but was mainly investigated at equilibrium [79]. Effects of multivalent particles [95] and multicomponent coexisting phases [127] were also explored. Dynamic investigation based on the Cahn–Hilliard equation has been recently suggested [128].

## 5. Conclusions and Outlook

Volume transition is a universal phenomenon identified in many synthetic and biopolymer gels. The steep response can be induced by incremental changes in multiple environmental parameters, such as ion concentration, ion valence, solvent quality, and temperature. The conformational change of polyelectrolyte molecules is accompanied by a steep change of certain physical properties of gels, such as their swelling degree, elastic modulus, magnetic relaxation rates, electric potential difference, ion partitioning, and water and ion apparent diffusion coefficients.

The nonlinear response of gels is an attractive characteristic, which is used in many industrial applications [129]. Their abundance in the natural world suggests that perhaps via the process of evolution different organisms have adopted this nonlinear response for their own benefit. Indeed, the phenomenon has been suggested to play an important role in various biological processes, including conformational changes of DNA molecules, cellular secretion of gel substances, control of water fluxes in the vascular tissue of plants, abrupt change in cell resting potential, and reversible condensation of many proteins and RNA molecules in the cytoplasm and nucleoplasm. Thus, important information about these systems may be derived from considerations of the physics of polyelectrolyte chains and their interactions with water molecules and ions.

Finally, the universality of the volume transition prompts the use of a hierarchical theoretical framework derived from first principles [3,4]. Macroscopic variables of the gel system, such as density, pressure, viscosity, and electric potential field, can be coupled with conservation laws and phenomenological constitutive relations. This allows us to associate the biological functions with trajectories in phase space, and provides a powerful means to understand, control, and manipulate the system. Nevertheless, microscopic details must be integrated into the mean-field framework and replace constants with functions accounting for dominant and specific molecular properties. Thus, it may be useful to connect molecular specificities of the biological systems to macroscopic variables of gels, in order to advance our understanding of state and emergent functionalities. 

## Abbreviations

NaPA	sodium polyacrylate
SANS	Small angle neutron scattering
SAXS	Small angle X-ray scattering
ADC	Apparent diffusion coefficient
